# Interspecific Neighbor Stimulates Peanut Growth Through Modulating Root Endophytic Microbial Community Construction

**DOI:** 10.3389/fpls.2022.830666

**Published:** 2022-03-03

**Authors:** Pin Chen, Wei He, Yi Shen, Lingyue Zhu, Xiangzhi Yao, Ruibo Sun, Chuanchao Dai, Bo Sun, Yan Chen

**Affiliations:** ^1^College of Life Sciences, Nanjing Normal University, Nanjing, China; ^2^State Key Laboratory of Soil and Sustainable Agriculture, Institute of Soil Science, Chinese Academy of Sciences, Nanjing, China; ^3^Institute of Industrial Crops, Jiangsu Academy of Agricultural Sciences, Nanjing, China; ^4^Anhui Province Key Laboratory of Farmland Ecological Conservation and Pollution Prevention, College of Resources and Environment, Anhui Agricultural University, Hefei, China

**Keywords:** interspecific facilitation, root-associated microbial assemblage, plant growth promotion, plant-microbial holobiont, low diversification

## Abstract

Plants have evolved the capability to respond to interspecific neighbors by changing morphological performance and reshaping belowground microbiota. However, whether neighboring plants influence the microbial colonization of the host’s root and further affect host performance is less understood. In this study, using 16S rRNA high-throughput sequencing of peanut (*Arachis hypogaea* L.) roots from over 5 years of mono- and intercropping field systems, we found that neighbor maize can alter the peanut root microbial composition and re-shape microbial community assembly. Interspecific maize coexistence increased the colonization of genera *Bradyrhizobium* and *Streptomyces* in intercropped peanut roots. Through endophytic bacterial isolation and isolate back inoculation experiments, we demonstrated that the functional potentials of available nutrient accumulation and phytohormones production from *Bradyrhizobium* and *Streptomyces* endowed them with the ability to act as keystones in the microbial network to benefit peanut growth and production with neighbor competition. Our results support the idea that plants establish a plant-endophytic microbial holobiont through root selective filtration to enhance host competitive dominance, and provide a promising direction to develop modern diversified planting for harnessing crop microbiomes for the promotion of crop growth and productivity in sustainable agriculture.

## Introduction

Intensive monoculture can meet the population demands for food production, however, this comes at the expense of belowground biodiversity and ecosystem functioning (Frison et al., 2011; Isbell et al., 2013). Low-diversity strategies such as legume/cereal, legume/grass intercropping provides a partial to complete substitute for some costly agricultural fertilizer inputs and positively impacts crop productivity and yield stability (Isbell et al., 2017; Tilman, 2020). Interspecific facilitation, from the perspective of resource heterogeneity and complementarity, has well explained the physiological performance of mutual promotion between interspecific species (Li et al., 2014; Fichtner et al., 2017; Schob et al., 2018; Zhang et al., 2021). However, in nature, plants do not live alone as single entities, but form a complex holobiont with microorganisms to adapt to various environmental conditions and changes (Niu et al., 2017; Zhang et al., 2019; Trivedi et al., 2020). These host dependent microbes have been reported to affect plant growth (Pieterse et al., 2014; Vandenkoornhuyse et al., 2015), but relatively few studies have sought to understand the effect of interspecific neighbors on focal plant root microbial colonization and their functioning in the field (Li et al., 2019; Gong et al., 2021). However, the dispersal effect of neighbor’s heterogeneous metabolic resources influenced the composition of the focal rhizosphere microbiota, which is the microbial resource pool of root endophytes (van der Heijden et al., 2008; Furey and Tilman, 2021). Simultaneously, plant belowground chemical and physical responses to non-kin recognition affect the host filtering of microbiome members (Zhalnina et al., 2018; Chen et al., 2020). These, theoretically, provide the possibility for focal plants to alter root microbial community construction.

Symbiotic microbiota are ubiquitous in the tissues of terrestrial plants (Fitzpatrick et al., 2018; Acuna-Rodriguez et al., 2020). Selection imposed by plant habitats strongly shapes the diversity and composition of symbiotic microbiota and leads to microbial adaptation associated with navigating the plant immune system, regulating plant growth and utilizing plant-derived resources (Hardoim et al., 2015; Foster et al., 2017; Fitzpatrick et al., 2020; Trivedi et al., 2020). Neighbor competition has been reported to induce the focal plant to selectively recruit rhizosphere microbial inhabitants (Zhang et al., 2019; Chen et al., 2020). It provides abundant microbial resources for the host to filter effective colonizers and unite them to increase host fitness (Ofek-Lalzar et al., 2014; Chen et al., 2018; Deyett and Rolshausen, 2020). Neighboring maize has been reported to result in the increased expression of genes mediating nodulation in the faba bean root, indicating higher colonization of dinitrogen fixing organisms (Li et al., 2016). In addition, two neighboring species *Deschampsia flexuosa* and *Trientalis europaea* share the dark septate endophyte *Phialocephala fortinii* in their roots for growth promotion (Tejesvi et al., 2013). These examples imply that plants have the potential to modulate microbial colonization to cope with neighbor co-existence.

The ability of specific endophytes to the synthesis of plant growth-promoting hormones (Hardoim et al., 2015; Vandana et al., 2021) and assist in the acquisition of additional resources (Asea et al., 1988; Rediers et al., 2003; Li et al., 2016; Xie et al., 2019; Chen et al., 2020) can increase microbial colonization efficiency when the host plant confronts to the variation of environmental factors (Navarro et al., 2006; Chen et al., 2017). Such changes in the relative abundance of individual species within a community lead to the re-assemblage of plant microbiome, and have large downstream effects on community composition and function (Fitzpatrick et al., 2018; Chen et al., 2020). The neighboring plant community constitutes an important component of a plant’s environmental and ecological context. Moreover, the neighborhood dispersal effect toward focal plant would increase as neighbor increase in biomass and age (Meyer et al., 2022). Whether the neighborhood effect could trigger plant host to shape root microbiota for improving host growth or fitness is remaining unknown.

To explore the effect of interspecific neighbors on the composition and function of the peanut (*Arachis hypogaea* L.) root symbiotic microbial community, we grew maize (*Zea mays* L.) and peanut, which are commonly co-cultivated in intensive agricultural systems (Li et al., 2007; Isbell et al., 2017). We used a high-throughput molecular approach (16S rRNA high-throughput sequencing of the peanut roots from over 5 years field systems, and back inoculation in soil experiments) and a culture-dependent approach (including endophytic bacterial isolation, and back inoculation experiments) into a single framework to answer the following questions: (1) Does the neighboring maize influence symbiotic colonization of the adjacent peanut root? If yes, (2) who initiates the change of the symbiotic community, and (3) how does the altered symbiotic community play a positive role in promoting host plant growth? We assumed that the plant “interspecific facilitation” effect may not be restricted only to plant-plant interaction, but could be expand to host-dependent microbiota, or at the very least to the root-associated microbiomes.

## Materials and Methods

### Mono- and Inter-Cropping Field Experimental Design

The field site was located at the Liuhe Plant Science Base of Jiangsu Academy of Agricultural Sciences, Jiangsu Province, China (32°36’N, 118°83’E). The site has a northern subtropical monsoon climate with a mean annual temperature of 15.6°C and a mean annual precipitation of 700–1900 mm. The frost-free period is 254 days. The soil type is classified as hydragric anthrosol (Wrb, 2014). In 2011, the soil of the experimental site contained organic matter (SOC) 12.12 g kg^–1^, nitrogen content 0.75 g kg^–1^, total phosphorus (TP) 0.53 g kg^–1^, total potassium (TK) 14.51 mg kg^–1^, soil nitrate nitrogen (NO_3_^–^-N) 10.69 mg kg^–1^, ammonium nitrogen (NH_4_^+^-N) 6.24 mg kg^–1^, available phosphorus (AP) 38.04 mg kg^–1^, and available potassium (AK) 225.83 mg kg^–1^, and had a pH of 6.97.

Three planting treatments were set up from 2012 to 2020: (1) peanut monocropping (PP); (2) maize monocropping (MM); and (3) maize/peanut intercropping (MP) (Supplementary Figure 1). In the PP treatment, the interrow and interplant distances were 0.85 and 0.2 m, respectively. In the MM treatment, the interrow and interplant distances were 0.85 and 0.4 m, respectively. The MP treatment included a 3.4 m peanut strip (four rows of peanut, with a 0.85 m interrow distance) and a 1.7 m maize strip (two rows of maize with 0.85 m interrow distance). The interplant distance within the same row was 0.2 m for peanut and 0.4 m for maize. Each plot was 8 × 5 m (length × width), and a ridge (with a width of 0.4 m and a height of 0.3 m) separated adjacent plots. Each treatment was conducted in triplicate plots.

In all treatments, the topsoil (0–25 cm depth) was plowed before cultivation every year. All plots received 120 kg ha^–1^ nitrogen fertilizer (urea containing 25% N), 75 kg ha^–1^ phosphorus (calcium superphosphate containing 30% P_2_O_5_), and 75 kg ha^–1^ K_2_O (potassium chloride containing 57% K_2_O). Peanuts were sown on 15–25 May and harvested on 15–25 September, while maize was sown on 15–25 June and harvested on 10–20 September. All plots were irrigated and weeded during the growing period. The yields of peanut were determined at harvest in September 2020.

### Plant Sampling in the Field

Field peanut plant samples were collected in PP and MP treatments at the peanut growing stage, while the maize plant samples were collected in MM and MP treatments at the jointing stages. Six peanut plants in each plot of PP and MP treatments were randomly selected for chlorophyll content determination *in situ* (SPAD 502 plus, Konica, Tokyo, Japan), and then collected for peanut growth characteristic indices and nutrient concentration determination. In addition, for peanut phytohormone, root microbiota detection and root cultivable isolation, peanut plants were cut and divided into the aboveground (leaf and shoot) and belowground (root) tissues. For the peanut aboveground tissue, the third and fifth leaves from the top of plant were selected. For the belowground tissues, peanut roots were washed until no visible soil particles remained. Then clean roots that were 1–2 cm down from the rhizome junction were cut and collected. Due to the small amount of biomass per plant sample, six peanut plants from each plot were selected for tissues collection and pooled into a single sample. In total, 24 peanut samples from the compositing of 144 peanut tissues [2 treatments (PP and MP) × 2 tissues (above- and underground tissues) × 2 replicates per plot (*n* = 6 for each) × 3 plot replicates] were collected from the field. All peanut aboveground samples were used for phytohormone determination. For peanut underground samples, each sample was divided into three parts: one part was frozen in liquid nitrogen for phytohormone determination, the second part was stored at -80°C for DNA extraction and molecular analysis, and the third was stored at 4°C for cultivable bacterial isolation. In parallel, 12 maize root samples composited from 72 maize roots [2 treatments (MM and MP) × 2 replicates per plot (*n* = 6 for each) × 3 plot replicates] were collected for endophytic microbial community determination. It should be noted that the endophytic microbial community treatments in our work included: PPpr, peanut root microbiota in peanut monocropping treatment; MPpr, peanut root microbiota in intercropping treatment; MMmr, maize root microbiota in maize monocropping treatment; MPmr, maize root microbiota in intercropping treatment.

### Endophytic Bacterial Isolation and Functional Detection

To explore the potential functions of the key root colonizers of intercropped peanut, we isolated endophytic bacteria from healthy intercropped peanut roots. The root samples were first washed with running water and then cut into small pieces (10 mm × 5 mm). Tissue pieces were surface sterilized using 75% ethanol and 3% sodium hypochlorite solution for 3 and 5 min respectively, and then washed five times with sterile distilled water (Schulz et al., 1993). The last water wash was incubated in LB medium to check whether the surface sterilization was complete (No colony growth indicating complete sterilization). The sterilized tissues were ground into homogenate, and diluted with 9 ml sterile water. Bacterial colonies were collected using Spread Plate Technique at the 10^–5^ dilution level. The media for the isolation was LB agar containing 10 g L^–1^ tryptone, 5 g L^–1^ yeast extract, 10 g L^–1^ NaCl, and 15 g L^–1^ agar, pH 7. Plates were placed at 30°C for 1–2 days. Single colonies that appeared on the plates were picked and purified using the streaking method, and pure isolates were then cultured in LB broth at 30°C on a shaker rotating at 220 rpm for 2 days for bacterial identification.

Bacterial genomic DNA was extracted using a Bacterial DNA Kit (Omega Bio-Tek, Inc., Norcross, GA, United States). The universal primers 27F (5’-AGAGTTTGATCCTGGCTCAG-3’) and 1492R (5’-GGTTACCTTGTTACGACTT-3’) (Lopez and Alippi, 2019), were used for PCR amplification (the protocol was consistent with PCR of 16S rRNA gene amplicon in Section “DNA Extraction and Bacterial 16S rRNA Gene Amplification”). The PCR products were then purified using a DNA Gel Extraction Kit (Axygen Bioscience, Inc., Union City, CA, United States) and sequenced by BGI Corp. (Shenzhen, China) for strain identification. The isolate identity was determined by the nucleotide BLAST of the sequence in GenBank of NCBI.^1^

Based on the construction of microbial co-occurrence network (see Section “Endophytic Microbial Network Construction”) and the result of Linear Discriminant Analysis coupled with Effect Size (LEfSe), we found that members of the genera *Bradyrhizobium* and *Streptomyces* acted as keystones of the root microbial network and were biomarkers in intercropped peanut. To select two representative strains of genera *Bradyrhizobium* and *Streptomyces*, bacterial isolates belonging to the two genera were detected for phytohormone production and nutrient transformation *in vitro*. Indole-3-acetic acid (IAA) production was tested using the method of cholorimetric assay with minimal medium supplemented with 1 g L^–1^ tryptophan (Loper and Schroth, 1986). Cytokinin production was determined using cytokinin (CTK) ELISA Kit (JiangLai Biotechnology, Shanghai, China). Bacterial potential dinitrogen-fixation and phosphate solubilization were qualitatively determined by diameter of transparent ring using Ashby’s nitrogen-free agar medium (nitrogen-free medium) and NBRIP (National Botanical Research Institute’s phosphate growth) medium (Nautiyal, 1999).

### Exogenous Inoculation of Bacterial Isolates in Plant Axenic and Soil Culture

**FIGURE 1:**
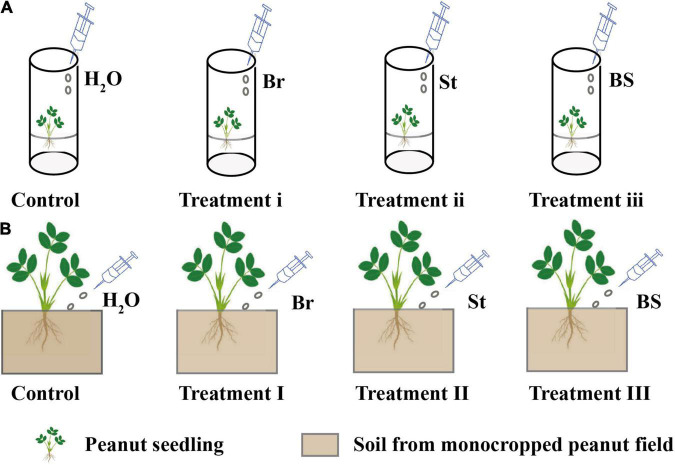
The exogenous inoculation of bacterial isolates in axenic and soil cultures. **(A)** Exogenous bacterial inoculation in axenic culture. **(B)** Exogenous bacterial inoculation in soil culture. For axenic and soil cultures **(A,B)**: Control (peanut inoculated with H_2_O); peanut inoculated with *Bradyrhizobium* EB56 in axenic (Treatment i) and soil culture (Treatment I); peanut inoculated with *Streptomyces* EB47 in axenic (Treatment ii) and soil culture (Treatment II) and peanut inoculated with mixed EB56 and EB47 in axenic (Treatment iii) and soil culture (Treatment III) were set up. Br, *Bradyrhizobium* EB56; St, *Streptomyce* EB47; BS, The combination of *Bradyrhizobium* EB56 and *Streptomyces* EB47.

To verify whether the specific bacterial isolates had a positive impact on peanut growth, inoculation experiments were performed in peanut axenic and soil culture. The experiments were conducted with three treatments in axenic and soil culture, respectively (Figures 1A,B): inoculation of *Bradyrhizobium* EB56 in axenic (Treatment i) and soil culture (Treatment I); inoculation of *Streptomyces* EB47 in axenic (Treatment ii) and soil culture (Treatment II); co-inoculation of *Bradyrhizobium* EB56 and *Streptomyces* EB47 in axenic (Treatment iii) and soil culture (Treatment III). Controls with water were processed identically. The two selected bacterial strains were inoculated in 25 ml of LB medium and incubated overnight at 37°C with shaking at 180 rpm. Bacterial cells were washed twice with sterilized water and cell suspensions were adjusted to 0.5 at OD_600_ for use as microbial agents before inoculation.

In plant axenic culture, surface sterilized peanut seeds were germinated and grown in Hoagland agar medium (100 ml) until seedling height reached 10–12 cm. For inoculation with individual isolates (Treatment i and ii), 1 ml of EB56 and EB47 microbial agent was injected around the peanut roots, respectively. For co-inoculation (Treatment iii), 1 ml of mixed culture (that included 0.5 ml of EB56 and 0.5 ml of EB47) was added around peanut roots. After inoculation, plants were placed in a growth chamber at a temperature of 25–28°C, 40–55% relative humidity, and a 12/12 h light/dark photoperiod. After 3 days, seedlings were collected for phytohormone determination. Each treatment was conducted for six replicates.

For soil cultivation, 100 g fresh soil (from 5 to 25 cm below the surface of peanut monocropping treatment in the field) was mixed and placed in a pot (height = 12 cm, diameter = 15 cm). Surface sterilized seeds were germinated in vermiculite for 3–5 days. Then the sprouted seedlings were transplanted to the pot. Meanwhile, 5 ml of EB56 or EB47 microbial agents were respectively sprayed around seedling roots for single isolate inoculations (Treatment I and II). For co-inoculation (Treatment III), 2.5 ml EB56 and 2.5 ml EB47 suspensions were mixed and then added to the seedlings’ rhizosphere. The soil culture conditions were consistent with the plant axenic culture. The soil pots were sealed and incubated at 25–30°C with watering every 2 days. After 25 days, plants were collected for growth and phytohormone determination. In addition, for peanut root samples, we also stored part of samples at −80°C for DNA extraction and PCR amplifications of the bacterial 16S rRNA genes (see this detailed method, refer to Section “DNA Extraction and Bacterial 16S rRNA Gene Amplification”). Each treatment was conducted with six replicates.

### Plant Growth and Nutrient Determination

The height and taproot length of five fresh peanut plants from each plot were measured. Each plant was then divided into above- and belowground tissues and the biomass weighed after drying at 65°C to a constant weight. Five gram biomass from each of the aboveground and underground dry samples were taken and pooled into a single sample for plant nutrient determination.

The plant nitrogen content was digested with sulfuric acid-perchloric acid (H_2_SO_4_-HClO_4_) (Nelson and Sommers, 1962). Plant phosphorus content and carbon content were determined by phosphorous vanadium molybdate yellow colorimetric method (Dick and Tabatabai, 1982) and potassium dichromate method, respectively.

### Plant Phytohormone Detection

Peanut phytohormone concentration was determined by phytohormone analysis (Balcke et al., 2012; Chen et al., 2020). Briefly, 100 mg of fresh plant material was ground into power in liquid nitrogen and extracted with 1.0 ml pre-chilled methanol: water: formic acid (7.9:2:0.1, v:v:v) overnight at 4°C. The suspension was centrifuged at 13,000 rpm for 20 min at 4°C, and the solid residue was re-extracted and re-centrifuged, the two supernatants were then pooled. The combined supernatant was passed through an Oasis MAX strong anion-exchange column (Waters, Milford, MA, United States) to remove interfering lipids and some of the plant pigments, and then dried under nitrogen gas. The residue was dissolved in 100 μl methanol.

The dissolved suspension was subjected to LC-MS/MS with an AB Sciex 5500 QTRAP spectrometer (AB Sciex, Toronto, Canada). The LC-MS/MS was operated in negative mode with electrospray as the ionization source. The separation was performed on a Waters ACQUITY HSS T3 (100 mm × 2.1 mm, 1.8 μm) column. Gradient elution was applied with a mobile phase of methanol and 0.1% aqueous formic acid at a flow rate of 0.3 ml min^–1^. The column temperature was maintained at 40°C, and the injection volume was 5 μl.

The calibration standards included a mixed phytohormone standard solution containing *trans*-zeatin (tZ), gibberellin A1 (GA1), gibberellin A3 (GA3), gibberellin A4 (GA4), abscisic acid (ABA), indole-3-acetic acid (IAA), salicyclic acid (SA), Brassinolide, jasmonic acid (JA), and methyl jasmonate (Me-JA) standards (Sigma-Aldrich, Saint Louis, MO, United States) at concentrations of 0.1, 1, 5, 10, 20, 40, 60, 80, and 100 ng ml^–1^ for each phytohormone standard in the mixed solution. The 1-aminocyclopropane-1-carboxylic acid (ACC) calibration standard was processed at the same concentration levels from 0.1 to 100 ng ml^–1^. The content of each phytohormone was calculated based on the standard curves in units of ng per mg fresh weight using Analyst software 1.6.

### DNA Extraction and Bacterial 16S rRNA Gene Amplification

Genomic DNA of root samples was extracted using Mag-Bind and Plant DNA KF 96 Kit (Omega, Norcross, GA, United States) according to the manufacturer’s instructions after surface sterilization following the procedure reported previously (Greweling, 1962). The quantity and purity of DNA were examined with a NanoDrop ND-1000 spectrophotometer (NanoDrop Technologies, Wolmington, DE, United States). The V5-V7 region of the root bacterial 16S rRNA gene was amplified using the primers 799F (AACMGGATTAGATACCCKG) and 1193R (ACGTCATCCCCACCTTCC) (Sofie et al., 2017). Each sample was amplified in a 50 μl reaction system with 20 μl water, 25 μl 2 × Premix Taq DNA polymerase (Takara, Kusatsu, Japan), 1 μl of each primer, 20 ng DNA templates. After an initial denaturation at 94°C for 5 min, the targeted region was amplified by 30 cycles of 94°C for 30 s, 52°C for 30 s, and 72°C for 30 s, followed by a final extension at 72°C for 10 min in a thermal cycler (GeneAmp PCR system 2700; Applied Biosystems, Caelsbad, CA, United States). Amplicon sequencing libraries were constructed using the MiSeq Reagent Kit v3 according to the manufacturer’s instructions. All samples were pooled in equimolar concentrations and then sequenced on the Illumina MiSeq platform with a paired-end protocol.

The raw sequencing data was processed using the QIIME pipeline (version 1.9.0) (Logares et al., 2012). To minimize the effects of random sequencing errors, low-quality and ambiguous reads (Phred quality score *Q* < 25 or sequence shorter than 150 bp) were eliminated. Chimeras were identified and removed with the UCHIME algorithm (Edgar et al., 2011). High-quality sequences were clustered into operational taxonomic units (OTUs) using UCLUST with a similarity threshold of 97%. The sequences were then phylogenetically assigned to taxonomic classifications using the RDP (Ribosomal Data Project database) classifier and were assigned at different levels (Wang et al., 2007). Singletons were removed, and all samples were rarefied to 55,000 sequences per sample for further analysis.

### Endophytic Microbial Network Construction

An ecological network is a representation of different biological interactions (competition, mutualism, etc.) in a given system, in which species (nodes) are connected by pairwise interactions (links) (Faust and Raes, 2012; Trivedi et al., 2020). In this study, we use these molecule-based ecological networks in microbial communities as molecular ecological network (MENs), in which different nodes (OTUs) are linked by edges (i.e., interactions) (Deng et al., 2012). The microbial networks were constructed based on bacterial OTU relative abundance in the field experiment. The MENs were constructed through Random Matrix Theory (RMT) based methods (Luo et al., 2007). The process of network construction has been integrated into a comprehensive and open-accessible Molecular Ecological Network Anasis Pipeline (MENAP) written in Java and Perlscripts (Deng et al., 2012).^2^ The covariations were determined across six biological replicates to create each network by constructing Pearson Rank correlations (*P* < 0.05). To reduce network complexity, we only considered bacterial genera with an average abundance > 1.5%. A threshold *S*_*t*_ can be defined as the transition of the nearest-neighbor spacing distribution of eigenvalues from GOE (Gaussian orthogonal ensemble) to Poisson distribution (Zhou et al., 2010). Various indices, including the average clustering coefficient (*avg*CC), average geodesic distance (GD), and size and modularity of the network, were calculated to describe network topologies (Deng et al., 2012). Average connectivity (*avg*K) was calculated to measure the complexity of the network structure (Xun et al., 2017). The topological role of each node was determined based on two properties: the within-module connectivity (*Zi*, how well a node is connected to other nodes in the same module) and the among-module connectivity (*Pi*, how well a node is connected to the nodes in other modules) (Guimera et al., 2007; Olesen et al., 2007). All nodes were sorted into four subcategories on the basis of these simple criteria: peripherals (nodes in the modules with few outside connections, *Zi* < 2.5 and *Pi* < 0.62), connectors (nodes that connect modules, *Pi* > 0.62), module hubs (highly connected nodes within modules, *Zi* > 2.5), and network hubs (highly connected nodes within the entire network, *Zi* > 2.5 and *Pi* > 0.62) (Olesen et al., 2007). The co-occurrence networks were visualized using Gephi software (version 0.9.2).

### Statistical Analysis

The differences in the peanut physiological traits in mono- and inter-cropping field systems were analyzed by an unpaired Mann–Whitney *U* test with GraphPad Prism v8.0 (GraphPad Software, Inc., La Jolla, CA, United States). The phylogenetic diversity index (alpha-diversity) and community dissimilarity were performed based on the rarefied OTU table using the vegan R package (Dixon, 2003; Hamady et al., 2009). Ordinary one-way analysis of variance (one-way ANOVA) by Tukey’s honest significant difference (HSD) test was performed to compare the alpha-diversity (Shannon, Evenness and Richness indices), the relative abundance of bacterial phyla and isolates’ functional detection. Principal-coordinate analysis (PCoA) was performed based on Bray-Curtis distances, and the coordinates were used to visualize differences in microbial community structure. Analysis of similarity (ANOSIM) and permutational multivariate analysis of variance (PERMANOVA) were performed to evaluate significant differences in microbial community composition among plant roots in different systems. The abundance-based β-null model was used to classify the relative influences of deterministic and stochastic process mediating community assembly (Tucker et al., 2015). The abundance-based β-null model was used to quantify the relative influences of distinct community assembly processes (Stegen et al., 2013). It was generated using 999 randomizations to obtain null expectations of community dissimilarities for each sample pair according to the framework described by Stegen et al. (2013). The null deviation value (NDV) is defined as the difference between the observed and averaged null dissimilarities. The NDV value close to 0 indicates higher influence of stochasticity, whereas NDV close to -1 or +1 indicates higher influence of deterministic processes structuring community assembly.

Linear discriminant analysis (LDA) coupled with effect size (LEfSe) is an algorithm that identifies features (genes, pathways, or taxa) characterizing differences between two or more biological conditions (Segata et al., 2011). Here, root bacterial taxa (genera) that differed significantly in terms of relative abundance in mono- and intercropped peanut roots were identified as potential biomarkers by LEfSe. Two treatment groups were used as the class of subjects. Taxa were identified at genus level using the following parameters: (1) alpha value = 0.05 for factorial Kruskal–Wallis tests among classes, and (2) threshold logarithmic LDA score > 2.0 for differential features. LEfSe is provide with a graphical interface in Galaxy framework,^3^ which allows users to select parameters to pipeline data between modules in a workflow framework, to generate publication quality graphical outputs.

## Results

### Neighboring Maize Affect Peanut Growth in the Field

When peanuts were co-cultivated with maize, plant height, root length, and biomass were 11.1, 62.8, and 22.0% higher than in monocropped peanut, respectively (*P* < 0.05; Table 1). As a result, intercropped peanut showed over 35.6% greater fruit weight (*P* < 0.0001). Along with the increase of peanut biomass, phosphorus content in intercropped peanut tissue was increased 26.2% than that in monocropped peanut (*P* < 0.05; Table 1). Although no difference was found in plant nitrogen and carbon contents between peanuts in the two cropping systems (*P* > 0.05), plant total carbon, nitrogen and phosphorus were increased (*P* < 0.001) due to the improvement of peanut biomass (Table 1).

**TABLE 1:** Peanut physiological characteristic in mono and inter-cropping treatments.*

Index	Monocropping system (PP)	Intercropping system (MP)	Mann–Whitney *U*	*P*
Plant height (cm)	21.74 ± 1.70	24.15 ± 1.54	13.50	**0.004**
Chlorophyll content	40.92 ± 3.98	43.62 ± 5.00	11.00	0.310
Root length (tapped root; cm)	12.26 ± 2.00	19.96 ± 3.93	1.00	**<0.001**
Aboveground biomass per plant (g)	0.48 ± 0.05	0.56 ± 0.07	14.00	**0.017**
Underground biomass per plant (g)	0.11 ± 0.02	0.16 ± 0.04	10.00	**0.006**
Fruit weight (g per plant^–1^)	10.06 ± 0.26	13.64 ± 0.53	0.00	**<0.0001**
C content (g kg^–1^)	354.30 ± 11.32	348.80 ± 13.87	55.00	0.347
N content (g kg^–1^)	20.97 ± 1.37	22.96 ± 3.97	24.00	0.442
P content (g kg^–1^)	2.25 ± 0.19	2.84 ± 0.49	20.00	**0.006**
Plant TC (mg plant^–1^)	206.20 ± 20.75	254.90 ± 27.51	5.00	**<0.001**
Plant TN (mg plant^–1^)	12.36 ± 1.54	16.37 ± 2.44	5.00	**<0.001**
Plant TP (mg plant^–1^)	1.30 ± 0.20	2.07 ± 0.35	4.00	**<0.001**

*TC, total carbon; TN, total nitrogen; TP, Total phosphorus.

*Values are the mean (n = 6) ± the standard deviation (SD) of the mean.*

*P values with bolding indicated the significant differences based on Mann–Whitney U test.*

**FIGURE 2:**
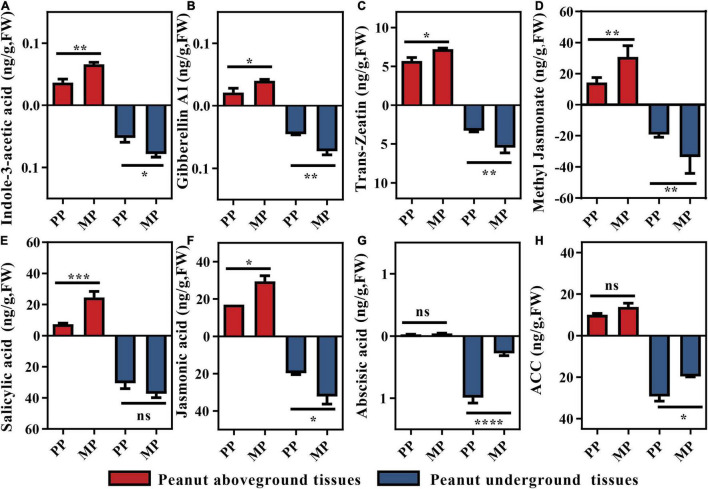
Detected phytohormone levels in peanut tissues. Statistical analyses were performed using Mann–Whitney *U* test. Significant difference is marked as follows: *, *P* < 0.05; **, *P* < 0.001; ***, *P* < 0.001; ns, not significantly different (*n* = 6). **(A)** Indole-3-acetic Acid; **(B)** Gibberellin A1; **(C)** *Trans*-Zeatin; **(D)** Methyl Jasmonate; **(E)** Salicylic acid; **(F)** Jasmonic acid; **(G)** Abscisic acid; **(H)** ACC, (1-aminocyclopropane-1-carboxylic acid). PP, peanut monocropping treatment; MP, peanut/maize intercropping treatment.

Plant physiological traits are often dependent on the changes in phytohormone level. Therefore, we assessed phytohormone status of both aboveground (including leaf and shoot) and belowground (including root) tissues (Figure 2 and Supplementary Figure 2). Consistent with the peanut physiological traits (Table 1), levels of growth promoting phytohormones including IAA, GA1 and tZ in intercropped peanuts showed at least 43.88% for aboveground tissues and 30.53% for underground tissues higher in intercropped peanuts than monocroped peanuts (*P* < 0.05; Figures 2A–C). Other three growth promoting phytohormones (GA3, GA4, and Brassinolide) showed the same tendency, but GA4 and Brassinolide in root were significantly different between treatments (Supplementary Figures 2A–C). Similarly, higher defense phytohormones including SA, JA, and Me-JA were found in above- and belowground tissues of intercropped peanuts than in monocropped peanuts, although the variance of SA in peanut roots was not significant (Figures 2D–F). However, the concentrations of ACC and ABA were average decreased over 16.46 and 68.60% in intercropped peanut roots, respectively (*P* < 0.05; Figures 2G,H).

### The Diversity and Composition of Peanut Root Microbiota

**FIGURE 3:**
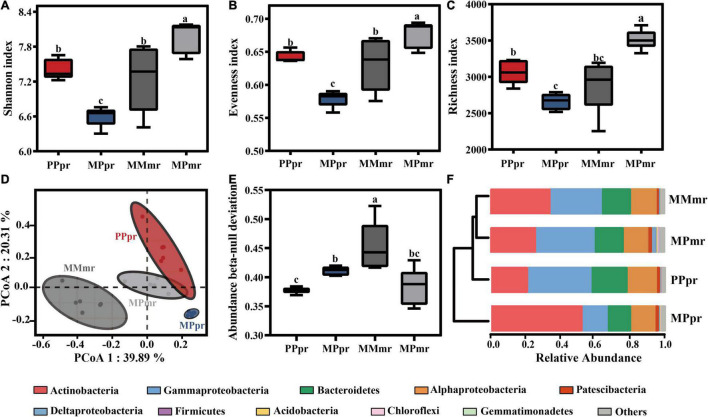
Alpha- and Beta-diversity of root endophytic microbiota in mono- and intercropping systems. Bacterial **(A)** Shannon index, **(B)** evenness index, and **(C)** Richness index of the root microbiota in mono- and inter-cropping treatments. Error bars are mean values ± SD (*n* = 6). Different letters indicate significant differences according to one-way analysis of variance (ANOVA) with Tukey’s HSD test (*P* < 0.05). **(D)** Principal coordinates analysis (PCoA) of root endophytic microbiota based on Bray-Curtis distances among different samples. **(E)** Abundance beta-null deviation used to indicate the difference of assembly of endophytic microbial communities. **(F)** Phylum-level distribution in the roots of peanut and maize in mono- and inter-cropping treatments. PPpr, peanut root microbiota in peanut monocropping treatment; MPpr, peanut root microbiota in intercropping treatment; MMmr, maize root microbiota in maize monocropping treatment; MPmr, maize root microbiota in intercropping treatment.

We used Shannon, Evenness, and Richness indices to evaluate the bacterial alpha-diversity of plant roots. Intercropping altered the root bacterial alpha-diversity in both plants, with intercropped peanut displaying the lowest diversity, whereas intercropped maize had the highest diversity (Figures 3A–C). No difference was found between the roots of monocropped peanut and maize (Figures 3A,B). Bray-Curtis distance in principle coordinate analysis (PCoA) was used to investigate beta-diversity of the root microbial community. As we expected, root microbial communities were clustered into four groups according to plant species and neighbor influence (Figure 3D). The separation of root microbiota in peanut was along the second principal component (PCoA2 = 20.31%; *P*_*PERMANOVA*_ = 0.004; Figure 3D; Supplementary Table 1). By comparison, root microbiota in maize were separated by cropping practice along the first principal component (PCoA1 = 39.89%; *P*_*PERMANOVA*_ = 0.005). The bacterial community cluster of intercropped maize was closer to the cluster of monocropped peanut root microbiota than that of monocropped maize (*R^2^* = 0.54 for monocropped maize and *R^2^* = 0.44 for intercropped peanuts; *P* < 0.05 for both; Supplementary Table 1). The NDV value of MPpr was higher than PPpr, indicating greater influence of deterministic (plant selection) processes on intercropped peanut (Figure 3E). In both peanut and maize roots, the bacterial phyla of *Actinobacteria*, *Gammaproteobacteria*, *Bacteroidetes*, and *Alphaproteobacteria* were present at relatively high abundance (average relative abundance > 10%; Figure 3F; Supplementary Figure 3). Among these dominant phyla, the relative abundance of *Actinobacteria* and *Acidobacteria* in intercropped peanut roots were increased, but *Gammaproteobacteria*, *Bacteroidetes*, and *Firmicutes* were reduced (*P* < 0.05; Supplementary Table 2). Additionally, intercropping resulted the lower abundance of *Actinobacteria*, but the higher abundance of *Deltaproteobacteria*, *Acidobacteria*, *Chloroflexi*, and *Gemmatimonadetes* in maize roots (*P* < 0.05; Supplementary Table 2). The relative abundance of endophytic *Alphaproteobacteria* was not affected by plant species or neighbor co-existence (*P* > 0.05; Supplementary Table 2).

### Endophytic Microbial Co-occurrence Networks in Peanuts Roots

**FIGURE 4:**
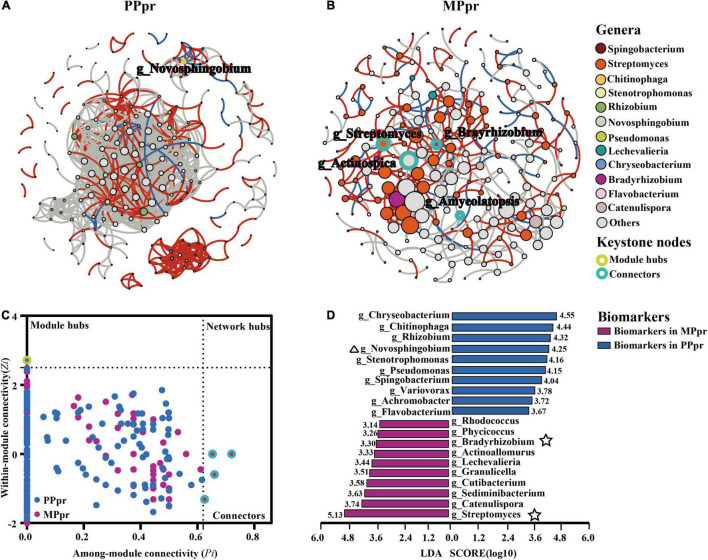
Bacterial co-occurrence network construction and biomarker detection. **(A,B)** Microbial networks of peanut root microbiota in mono- and inter-cropping treatments. A connection indicates a strong (The threshold for Person > 0.8) and significant (*P* < 0.01) correlation in PPpr **(A)** and MPpr **(B)** networks. Nodes representing the biomarker genera discovered by LEfSe in both mono- and inter-cropping peanut roots are colored according to genus, and other nodes are colored gray. The size of the nodes shows the abundance of genera. To distinguish module hubs in PPpr and connectors in MPpr, nodes that represent module hubs and connectors are highlighted with yellow and light blue circles, respectively. Edges directly connected to biomarker genera are colored: a red edge indicates positive interactions between two individual nodes, while a blue edge indicates negative interactions. **(C)** *Zi-Pi* plot showing the distribution of core genera based on their topological roles the monocropping **(A)** and intecropping **(B)** networks. Each symbol represents a genus in the bacterial network. The within-*Zi* and among-*Pi* module connectivity plot was used to identify module hub (*Zi* > 2.5, *Pi* ≤ 0.62) or connectors (*Zi* ≤ 2.5, *Pi* > 0.62) in the networks. The symbols that represent module hubs and connectors are also highlighted with yellow and light blue circle, respectively. **(D)** The bacterial biomarkers at genus level in mono- and inter-cropped peanut roots according to the linear discriminant analysis (LDA) scores of Linear discriminant analysis coupled with Effect Size (LEfSe). The top 10 representative biomarkers in each treatment are shown at genera level. Triangle and star symbols next to biomarkers indicate that those genera were also microbial keystones in PPpr and MPpr co-occurrence networks, respectively.

We constructed co-occurrence networks to determine the differences of bacterial potential interactions in peanut roots. Overall, crop planting showed a marked effect on the endophytic microbial network: the average clustering coefficient (*avg*CC), average path distance (GD) and modularity indices of the empirical networks were larger than those of their respective identically sized random networks (Table 2). The graph density (D), average degree (*avg*K), and *avg*CC showed lower values in intercropped peanut root (MPpr) than that in monocropped peanut root (PPpr), indicating intercropping resulted in a simpler bacterial co-occurrence network in peanut roots (Figures 4A,B and Table 2). Having neighboring maize resulted in a strong decrease of positive edges, and a slight increase of negative edges in the intercropped peanut symbiotic community (Figures 4A,B and Table 2). As a result, we observed that the ratio of positive to negative edges in MPpr was nearly nine times as much as in PPpr (Table 2). We then used within- (*Zi* degree) and among-module (*Pi* degree) connectivity to identify important nodes (representing OTUs) that may act as keystones in the microbial networks. Five keystones including one module hub and four connectors were identified in mono- and inter-cropped peanut root networks, respectively (Figure 4C and Supplementary Table 3). In PPpr co-occurrence network, the module hub (OTU114) was affiliated with *Novosphingobium*, which belongs to the phylum *Alphaproteobacteria*. In MPpr co-occurrence network, the four connectors (OTU3045, OUT454, OTU1222, and OTU223) were affiliated with the genera of *Bradyrhizobium*, and *Streptomyces*, *Actinospica*, and *Amycolatopsis*, which belong to the phyla *Alphaproteobacteria* and *Actinobacteria*, respectively (Figure 4C and Supplementary Table 3).

**TABLE 2:** Topological properties of the empirical molecular ecological networks of root microbiota in treatments.*

Network metrics	PPpr	MPpr
**Empirical networks**		
Similarity threshold (st)	0.96	0.95
Number of nodes	249	231
Number of edges	1317	408
Number of positive correlations	1265	299
Number of negative correlations	52	109
Ratio of positive to negative	24.33	2.74
Average degree (*avg*K)	10.58	3.53
Average clustering coefficient (*avg*CC)	0.41	0.35
Average path distance (GD)	5.32	7.07
Geodesic efficiency (E)	0.32	0.27
Centralization of degree (CD)	0.15	0.04
Density (D)	0.04	0.02
Modularity	0.46	0.76
**Random network**		
Average clustering coefficient (*avg*CC)	0.15 ± 0.010	0.02 ± 0.006
Average path distance (GD)	2.78 ± 0.031	4.25 ± 0.077
Modularity	0.22 ± 0.006	0.54 ± 0.008

*PPpr, peanut root microbiota in monocropping treatment; MPpr, peanut root microbiota in intercropping treatment.

Using Linear Discriminant Analysis coupled with Effect Size (LEfSe), a total of 68 biomarker genera were detected in the endophytic communities of mono- (45 biomarkers) and intercropped (23 biomarkers) peanut roots (Supplementary Figure 4). Among the top 10 representative biomarker genera (LDA log score threshold > 2.0 and *P* < 0.05), the genus *Novosphingobium* (g_Novosphingobium OTU114, acted as a module hub in the PPpr microbial network, Figure 4A) was ranked fourth (marked with triangle symbol) in PPpr (Figure 4D), and the genera *Streptomyces* and *Bradyrhizobium* (g_Streptomyces OTU454 and g_Bradyrhizobium OTU3045, acted as connecters in MPpr microbial network, Figure 4B) were ranked first and eighth (marked with star symbol) in MPpr (Figure 4D).

### Isolation and Functional Detection of Intercropped Peanut Root Bacteria

**FIGURE 5:**
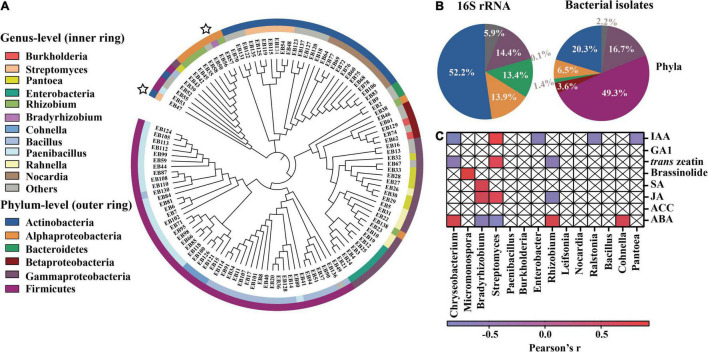
Information of cultivable bacteria isolated from intercropped peanut roots. **(A)** Cladogram showing phylogenetic relationships between the 117 heterotrophic bacterial isolates. Leaf labels represent representative sequence IDs. The inner circle indicates the genus-level taxonomy of isolates. The outer circle indicates the phylum-level taxonomy of isolates. **(B)** Pie charts show the proportion of different phyla from the 16S rRNA gene high-throughput sequencing data (left) and bacterial isolates (right). Colors in pies represent the percentage of OTUs (left) and isolates (right) at phylum level. **(C)** The Spearman correlation Heatmap of the relatively abundant core genera in16S rRNA sequencing (those that were consistent with the genera of the isolates) in relation to the phytohormone concentrations in intercropped peanut roots. The relationships were calculated based on Spearman correlation. The color from blue to red represents the relations of the relative abundance core genera and the phytohormone concentrations from negative to positive (the Pearson’s *r* value range of −1 to 1, *P* < 0.05). The box with a cross represents the relative abundance of those genera that had insignificant correlation with phytohormone concentrations.

To confirm the potential functions of specific genera in peanut roots, we isolated 117 endophytic bacterial strains from the intercropped peanut roots (Figure 5A). These isolates mainly belonged to five phyla, including *Firmicutes* (49.3%), *Actinobacteria* (20.3%), *Gammaproteobacteria* (16.7%), and *Alphaproteobacteria* (6.5%), which were partly consistent with the dominant phyla (such as *Actinobacteria*, *Gammaproteobacteria*, and *Alphaproteobacteria*) observed in the 16S high-throughput sequencing data (Figure 5B). The main difference was the high proportion of the phylum *Firmicutes* being isolated, which may due to the specific medium selection. According to the phylogenetic classification, 14 genera of the isolates can be matched with high-throughput sequencing. We then use Pearson’s r to find genera that can potentially promote plant growth. Nine of those matched genera showed significant correlation with at least one phytohormone (Figure 5C). It is worth noting that the genera *Streptomyces* and *Bradyrhizobium*, again, showed positive relations to plant growth promoting (IAA and tZ) and/or defense (SA and JA) phytohormones, but negative relation to ABA (Figure 5C).

Based on the result of microbial network and bacterial potential function selection, we targeted the two genera *Streptomyces* and *Bradyrhizobium* for the follow-up verification. In total, we had eight isolates belonging to *Streptomyces* and a single isolate belonging to *Bradyrhizobium* (Supplementary Table 4). All of these isolates could produce IAA and cytokinin (Supplementary Figures 5A,B) and were able to grow in nitrogen-free medium and phosphate solubilizing medium (NBRIP) (Supplementary Figures 5C,D). Among the 8 *Streptomyces* isolates, strain EB47 showed the highest IAA and cytokinin production, and a relatively high capability for P mobilization (Supplementary Figure 5D). Therefore, strains *Streptomyces* EB47 and *Bradyrhizobium* EB56 were selected for further incubation experiments.

### Exogenous Inoculation of Bacteria Promotes Peanut Growth

**FIGURE 6:**
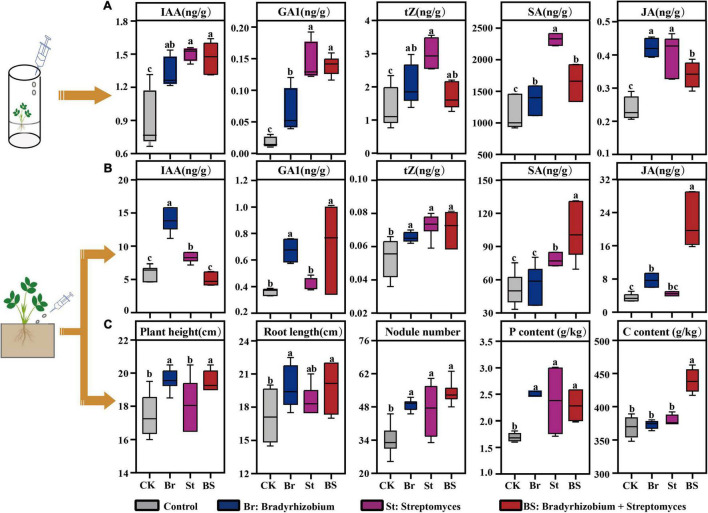
Effect of exogenous inoculation of bacterial isolates on peanut growth. **(A)** Detected phytohormone levels of peanut seedling in axenic culture with exogenous inoculation of bacterial isolates. **(B)** Phytohormone levels of peanut seedling in soil culture after exogenous inoculation of bacterial isolates. **(C)** Seedling physiological characteristic in soil culture after exogenous inoculation of bacterial isolates. Error bars in box are mean values ± SD (*n* = 6). Different letters above the error bars indicate significant differences according to one-way analysis of variance (ANOVA) with Tukey’s HSD test (*P* < 0.05). IAA, indole-3-acetic acid; GA1, gibberellin A1; tZ, *trans*-zeatin; SA, salicylic acid; JA, jasmonic acid; CK, Control; Br, peanut seedlings inoculated with *Bradyrhizobium* EB56; St, peanut seedlings inoculated with *Streptomyces* EB47; BS, peanut seedlings inoculated with EB56 and EB47.

Both in axenic and soil cultures, exogenous bacterial inoculation influenced peanut growth promoting and defensive hormone production (Figures 6A,B). The majority of phytohormone responses by axenic seedling to individual bacterial exogenous inoculations, especially *Streptomyces*, were stronger than for co-inoculation (*P* < 0.05; Figure 6A). However, when peanuts were grown in soil, the plants showed high hormone feedback to co-inoculation (*P* < 0.05; Figure 6B), except for IAA production (*P* > 0.05; Figure 6B). As a result, plant physiological characteristics including plant height, root length, and nodule numbers inoculated with the isolates were significantly higher than in the Control (Figure 6C), although the plant biomass did not display any significant difference (Supplementary Figures 6A,B). In parallel, the content of plant carbon, nitrogen, and phosphorus were higher in co-inoculation treatment compared with Control (Figure 6C and Supplementary Figure 6C). These results from the soil pot experiment were consistent with intercropped peanut growth and hormone response in the field (Figure 2 and Table 1).

**FIGURE 7:**
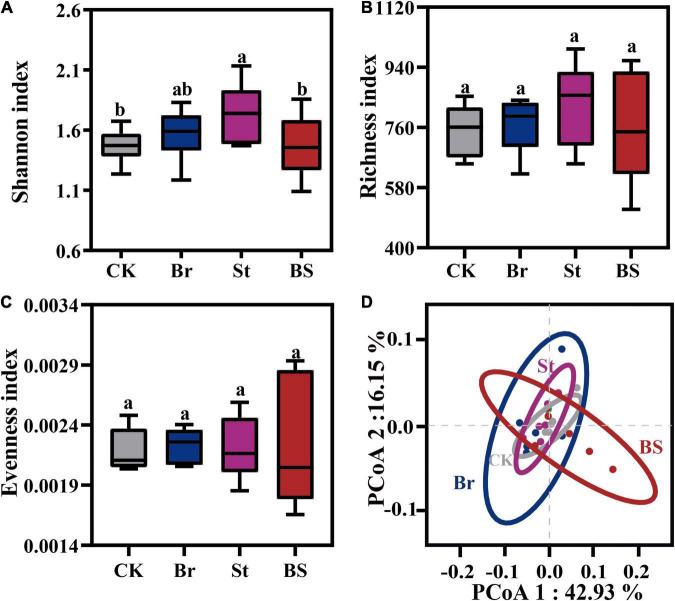
The exogenous bacterial stimulation effect on peanut root endophytic microbiota. Bacterial **(A)** Shannon, **(B)** Richness, and **(C)** evenness index of the root endophytic microbiota from single and co-inoculation treatments. Error bars in columns are mean values ± SD (*n* = 5). Different letters above the error bars indicate significant differences according to one-way analysis of variance (ANOVA) with Tukey’s HSD test (*P* < 0.05). **(D)** Principal coordinates analysis (PCoA) of root endophytic microbial community based on Bray-Curtis distances among different treatments. Br, Bradyrhizobium EB56; St, Streptomyce EB47; BS, The combination of Bradyrhizobium EB56 and Streptomyces EB47.

Additionally, we also investigated the microbial community that colonized peanut roots after exogenous inoculation. Interestingly, bacterial inoculation had no effect on microbial alpha-diversity (*P* > 0.05; Figures 7A–C). Consistently, root microbial compositions of individual- and co-inoculation treatments showed overlapping clusters (*P*_*PERMANOVA*_ > 0.05; Figure 7D; Supplementary Table 5). That means exogenous bacterial inoculation could temporarily promote plant growth, but have no persistent influence on root microbial colonization.

## Discussion

### Peanut Physiological Responses When Neighbored With Maize

As a sustainable agricultural practice, legume-cereal intercropping has been widely reported to support nutrient cycling and crop yield (Fan et al., 2006; Xu et al., 2020). Consistent with previous studies, we found that intercropping with maize resulted greater nutrient acquisition in the intercropped peanut (Table 1). Li et al. (2007, 2016) demonstrated that the release of heterogeneous root exudates between legumes and maize could promote dinitrogen-fixation and mobilize soil phosphorus for host acquisition (Li et al., 2007; Li et al., 2016). The more nutrient acquisition for intercropped peanut, the higher focal peanut biomass and fruit weights (Table 1). Phytohormones play central roles in controlling plant performance during plant development (Eichmann et al., 2021). In line with peanut growth in the field, intercropped peanuts showed higher levels of growth-promoting phytohormone (including IAA, GA1, and tZ) in tissues. It is well known that IAA, GA, and tZ regulate root development, aboveground elongation and plant cell division, respectively (Hedden and Sponsel, 2004). Therefore, the increased level of these phytohomones would be the important inner factor enhancing peanut productivity. Simultaneously, the defense hormones (including SA, JA, and Me-JA) in intercropped peanut tissues (Figure 2) were increased, indicating that interspecific neighboring plants stimulated the defense system of the focal peanut for environmental adaptation.

### Neighboring Maize Altered Peanut Root Microbial Composition and Assemblage

In addition to host self-programmed physiological metabolism, microbial invasion and colonization also alter host phytohormone expression and plant performance (Shropshire and Bordenstein, 2016; Zhang et al., 2020). Intra- and interspecific neighbors drive local plant rhizosphere microbiota to converge and diverge, respectively (Chen et al., 2020; Kong et al., 2021). In this study, we observed that neighboring maize enhanced the deterministic processes by habitat filtering of peanut (Figure 3E). Such enhanced root selection resulted lower alpha-diversity of the intercropped peanut root microbiota (Figures 3A–C). Interestingly, the response of maize root to interspecific neighbor was completely opposite to that of peanut (Figures 3A–C), indicating the filtering strategies of microbial community differed between plant genotypes (Lundberg et al., 2012; Zhang et al., 2019; Wagner et al., 2020; Xiong et al., 2021). In addition, we found that replicates of microbial composition of MPpr clustered closer than that of PPpr, and even maize root microbiota of MPpr clustered more closely with peanut (PPpr and MPpr) than MMmr (Figure 3D). Our previous study demonstrated that peanut with competitor stress can produce gaseous ethylene belowground (Chen et al., 2020). These gaseous signals have a wide diffusion, and therefore effect, range in soil and may lead to the colonization of relatively homogenous endophytic communities both in peanut and maize roots (Schmidt et al., 2019; Chen et al., 2020).

### Neighboring Maize Simplified Microbial Co-occurrence Networks in Peanuts Roots

Microorganisms within a community are not isolated, but form complex webs of ecological interactions (Faust and Raes, 2012; Trivedi et al., 2020). Microbial co-occurrence network analysis based on computational methods provides a promising approach to investigate various types of associations and identify potential keystone organisms within a given microbial network (Agler et al., 2016; Shi et al., 2016; Trivedi et al., 2017). In this study, the intercropped peanut root bacterial assemblage formed a less connected and simpler network compared with monocropped peanut (Figure 4 and Table 2). Xiong et al. demonstrating that the strength of host selection determines the microbial diversity and network complexity (Xiong et al., 2021). Due to maize competition, the peanut exhibited a stronger selection of microbiota in roots, thereby resulting in lower network complexity (Figures 3A–C, 4A,B). Within the microbial network, the “core” microbiome (including module hubs and connectors) plays a role in mediating microbial assembly (Zhou et al., 2010; Banerjee et al., 2018). Among the four keystones of the intercropped peanut microbial network (Figures 4B,C), *Bradyrhizobium* had been demonstrated to fix dinitrogen (Rouws et al., 2014). Its importance in the assembly of soil microbial communities has been widely reported (Etalo et al., 2018; Han et al., 2020), possibly because of their ability to act as a nutrient resource supply for microbial consumers. *Streptomyces*, *Actinospica*, and *Amycolatopsis* belong to *Actinomycetia*, which is a bacterial class that has been a source of highly diverse antibiotics (Gordon et al., 1962). Natural antibiotic production may have led to the increase of negative associations in the intercropped peanut root (Figure 4B and Table 2). Similar species within the same class (*Actinomycetia*) that participated in reshaping microbial communities were also observed in the rhizosphere of intercropped peanut (Chen et al., 2020). While in monocropped peanut root, *Novosphingobium* was the keystone for microbial community construction (Figures 4A,D). *Novosphingobium* is reported to be a class of microorganisms that can decompose organic matters containing benzene rings. The accumulation of allelochemicals (e.g., phenolic acids) around monocropped peanut root may cause the enrichment of *Novosphingobium* (Huang et al., 2013).

### Bacterial *Bradyrhizobium* and *Streptomyces* Promote Peanut Growth

Host plants establish mutual symbiotic relations with colonizers to improve environmental adaptability (Vandenkoornhuyse et al., 2015; Zhalnina et al., 2018; Newman and Derbyshire, 2020). Here, using bacterial inoculation experiments, we found that two important genera, *Bradyrhizobium* and *Streptomyces*, acted as biomarkers and keystones in intercropped peanut (Figure 4). Consistent with Chaparro et al. (2014), these beneficial genera were also found as key connectors in the network during host plant development when no external fertilization was applied to the field. The isolates of these two genera can produce plant growth hormones and increase P mobilization *in vitro* (Supplementary Figures 5A,B,D). Although their N_2_ fixing capabilities needed to be confirmed by N_2_-fixing gene (such as *nif* genes) demonstration and acetylene reduction, their growth in nitrogen free medium (Supplementary Figure 5C) suggest possible non-symbiotic dinitrogen fixation activity. Therefore, host root colonization by these beneficial microbes may not only expand the channels for plants to obtain nutrients, but help the plants to adjust hormone levels for growth and production. In this study, neighboring maize induced more colonization of peanut roots by these species. This may be attributed to maize-specific secretions, as Li et al. (2016) demonstrated that maize root exudates up-regulate key nodulation genes of legumes, while wheat root exudates cannot. Our results also implicated the importance of neighbor plant identity in focal plant selection of root endophytic colonization. Additionally, it is worth noting that, although exogenous functional bacteria application can increase peanut phytohormone level and improve host physiological characteristics (Figure 6), it cannot cause the alteration of the microbial community that colonized the peanut roots. This implies we can’t improve the host colonization of these beneficial bacteria through a simple high-dose exogenous inoculant application. Because that the niche of exogenous bacteria in the soil is often outcompeted by other dominant microbial species. Without specific neighboring effect, the habitats (e.g., soil) of these bacteria are easily occupied by indigenous microorganisms (Niu et al., 2021), resulting in unsustainable plant growth promotion. Many studies have confirmed that exogenous functional bacteria were at a niche disadvantage when competing with indigenous soil microbiota, leading to their extinction. This is also a challenge for the development of soil microbial agent application. Our study supported that using interspecific plant neighboring effect to alter bacterial community colonization and optimize host fitness at the “plant-microbiome” holobiont level, would be a new path for sustainable agricultural development (Trivedi et al., 2021).

## Conclusion

In this study, we found that neighboring maize promoted the growth and yield of the focal peanut by modulating peanut root endophytic microbial community composition and assembly. Interspecific neighbors induced peanut colonization by a higher abundance of *Bradyrhizobium* and *Streptomycetes*, both of which are capable of producing growth promoting phytohormones and mobilizing P resource. These genera also acted as keystone organisms in endophytic microbial network of the intercropped peanut root. Exogenous inoculation of these bacteria benefited plant growth by elevating growth promoting and defense hormones and increasing nutrient accumulation. However, the inoculation could not alter root microbial colonization. Our study provides a promising new direction for targeted manipulations of the root-associated microbiome through reasonable intercropping strategies in intensive agricultural systems.

## Data Availability Statement

The datasets presented in this study can be found in online repositories. The names of the repository/repositories and accession number(s) can be found below: https://www.ncbi.nlm.nih.gov/genbank/, MZ196225 to MZ196331; https://www.ebi.ac.uk/ena, BioProject ID PRJNA732240.

## Author Contributions

YC, WH, BS, and CD designed the research. PC, YC, YS, and XY performed experiments and conducted fieldwork. YC, PC, RS, and LZ analyzed the data. YC and PC wrote the manuscript. All authors read and approved the final manuscript.

## Conflict of Interest

The authors declare that the research was conducted in the absence of any commercial or financial relationships that could be construed as a potential conflict of interest.

## Publisher’s Note

All claims expressed in this article are solely those of the authors and do not necessarily represent those of their affiliated organizations, or those of the publisher, the editors and the reviewers. Any product that may be evaluated in this article, or claim that may be made by its manufacturer, is not guaranteed or endorsed by the publisher.

## Footnotes

1
https://www.ncbi.nlm.nih.gov/

2
http://ieg4.rccc.ou.edu/mena/

3
http://huttenhower.sph.harvard.edu/galaxy
