# Supplementary material for: Interspecific Neighbor Stimulates Peanut Growth Through Modulating Root Endophytic Microbial Community Construction
Source: Front Plant Sci. 2022 Mar 3;13:830666. doi: 10.3389/fpls.2022.830666 (PMC8928431; doi:10.3389/fpls.2022.830666)
Supplement: Supplementary file 1 [file Image_1.PDF]

Supplementary Information

Supplementary Figures

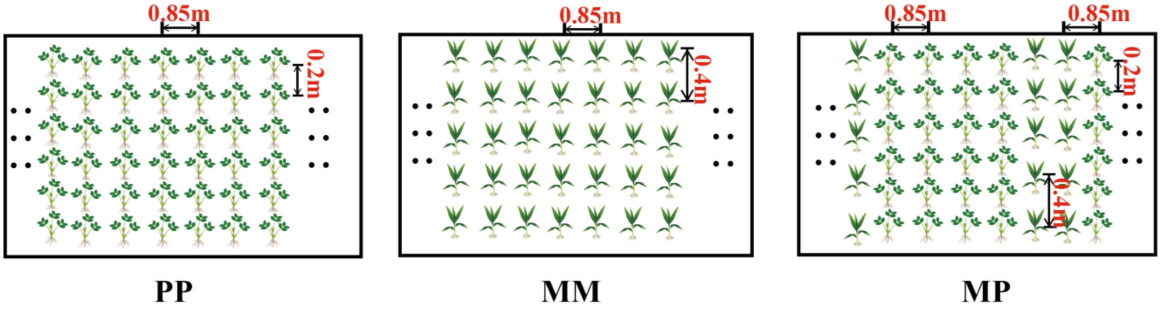

Supplementary Figure 1. The schematic of mono- and inter-cropping field experiment. The field experiment of peanut monocropping (PP), maize monocropping (MM) and peanut/maize intercropping (MP) treatments.
