# Supplementary material for: Interspecific Neighbor Stimulates Peanut Growth Through Modulating Root Endophytic Microbial Community Construction
Source: Front Plant Sci. 2022 Mar 3;13:830666. doi: 10.3389/fpls.2022.830666 (PMC8928431; doi:10.3389/fpls.2022.830666)
Supplement: Supplementary file 2 [file Image_2.PDF]

Supplementary Information

Supplementary Figures

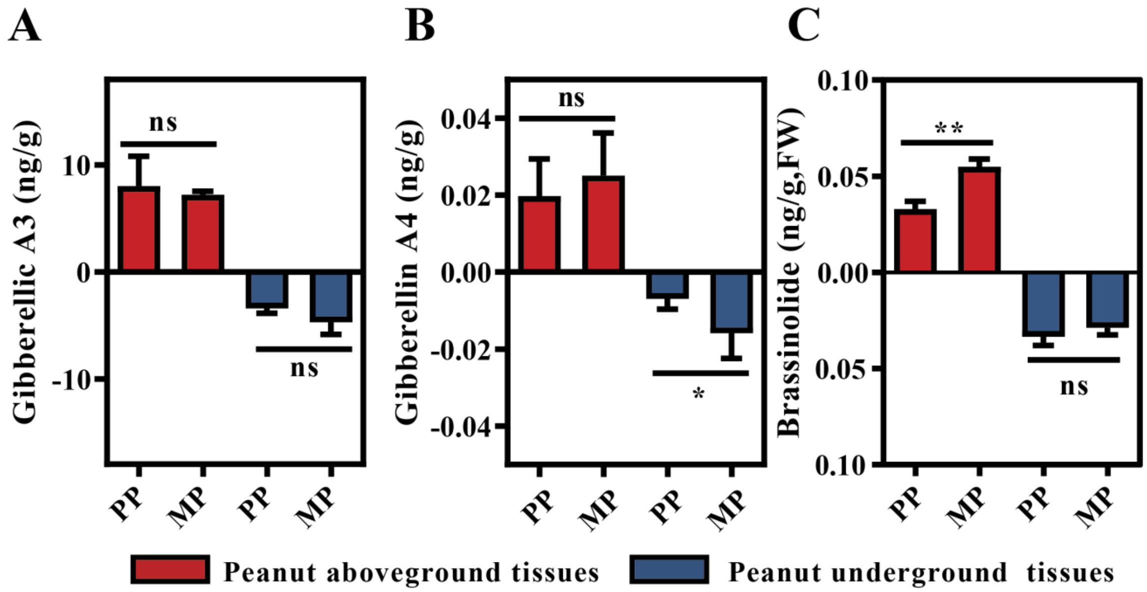

Supplementary Figure 2. Detected phytohormone levels in peanut tissues, including gibberellin A3 (A), gibberellin A4 (B), Brassinolide (C). Statistical analyses were performed using Mann-Whitney *U* test. Significant difference is marked as follows: *, *P*<0.05; **, *P*<0.001; ***, *P*<0.001; ns, not significantly different (n=6).
