# Supplementary material for: Interspecific Neighbor Stimulates Peanut Growth Through Modulating Root Endophytic Microbial Community Construction
Source: Front Plant Sci. 2022 Mar 3;13:830666. doi: 10.3389/fpls.2022.830666 (PMC8928431; doi:10.3389/fpls.2022.830666)
Supplement: Supplementary file 3 [file Image_3.PDF]

Supplementary Information

Supplementary Figures

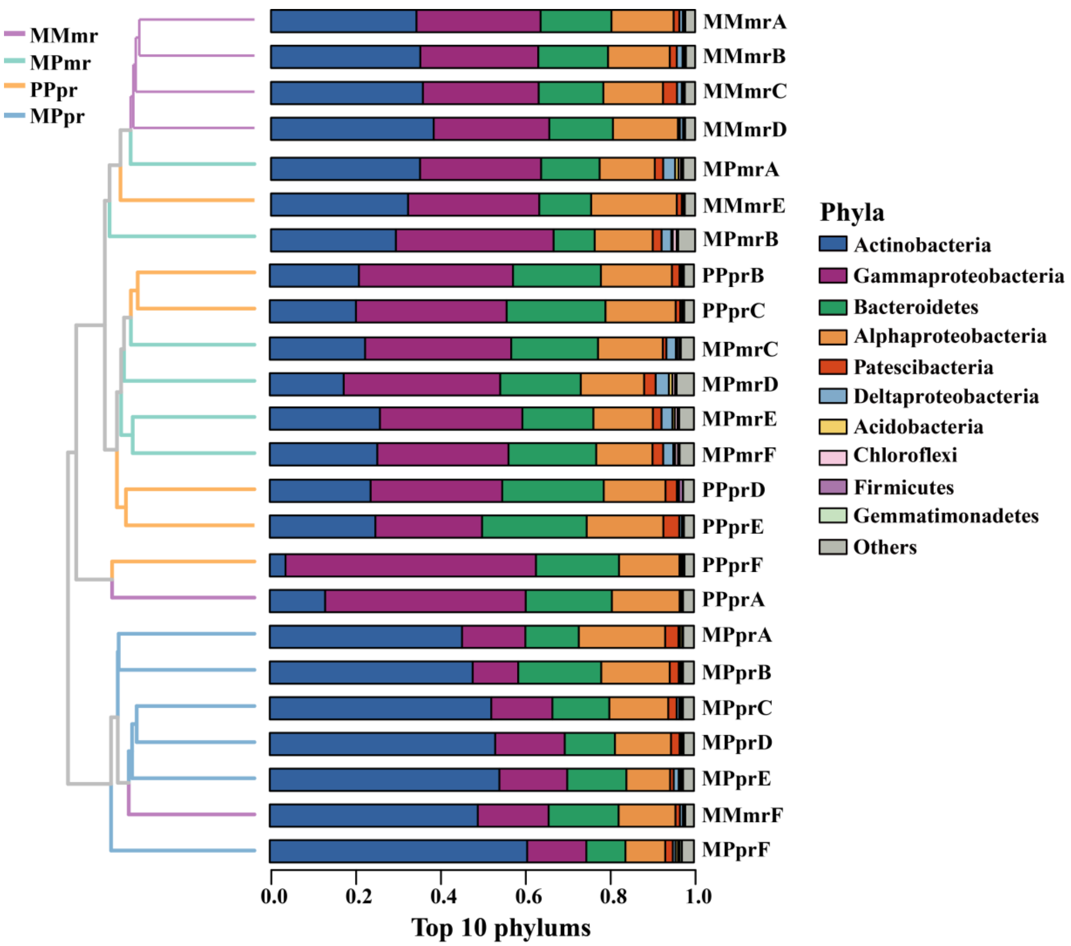

Supplementary Figure 3. Phylum-level distribution of microbial composition in the different cropping systems. PPpr, peanut root microbiota in monocropping treatment; MPpr, peanut root microbiota in intercropping treatment; MMmr, maize root microbiota in monocropping treatment; MPmr, maize root microbiota in intercropping treatment.
