# Supplementary material for: Interspecific Neighbor Stimulates Peanut Growth Through Modulating Root Endophytic Microbial Community Construction
Source: Front Plant Sci. 2022 Mar 3;13:830666. doi: 10.3389/fpls.2022.830666 (PMC8928431; doi:10.3389/fpls.2022.830666)
Supplement: Supplementary file 4 [file Image_4.PDF]

Supplementary Information

Supplementary Figures

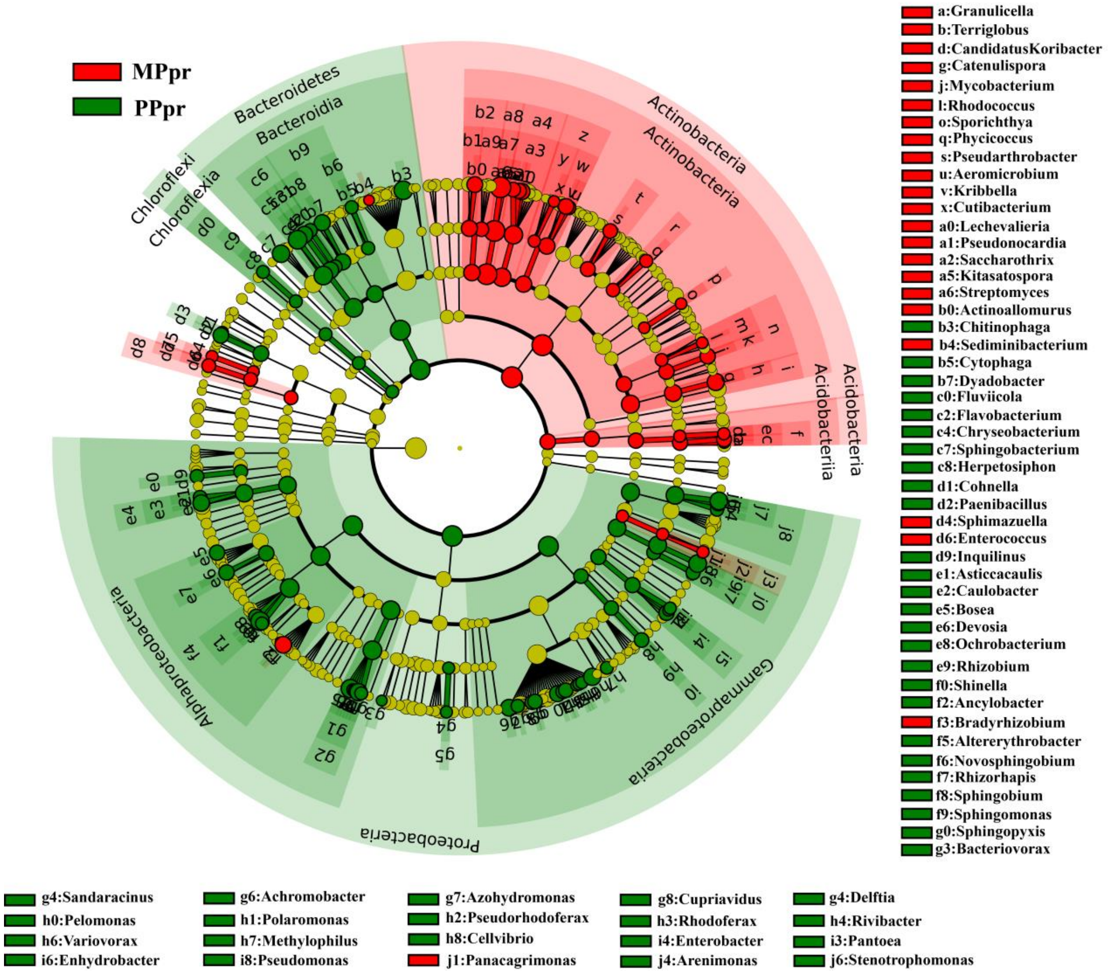

Supplementary Figure 4. Linear discriminant analysis (LDA) coupled with effect size (LEfSe) identifying potential biomarkers of bacterial in monocropped and intercropped peanut root. Bacterial biomarkers significantly differing in their relative abundance among treatments are indicated by colored dots, and from the center outward, they represent the kingdom, phylum, class, order, family, and genus levels. Colored shading represents the trend of distinctly different taxa. Only taxa meeting an LDA threshold of >2 are shown. Biomarkers ranking top 10 in each treatment group were listed in **Figure 4D**.
