# Supplementary material for: Interspecific Neighbor Stimulates Peanut Growth Through Modulating Root Endophytic Microbial Community Construction
Source: Front Plant Sci. 2022 Mar 3;13:830666. doi: 10.3389/fpls.2022.830666 (PMC8928431; doi:10.3389/fpls.2022.830666)
Supplement: Supplementary file 5 [file Image_5.PDF]

Supplementary Information

Supplementary Figures

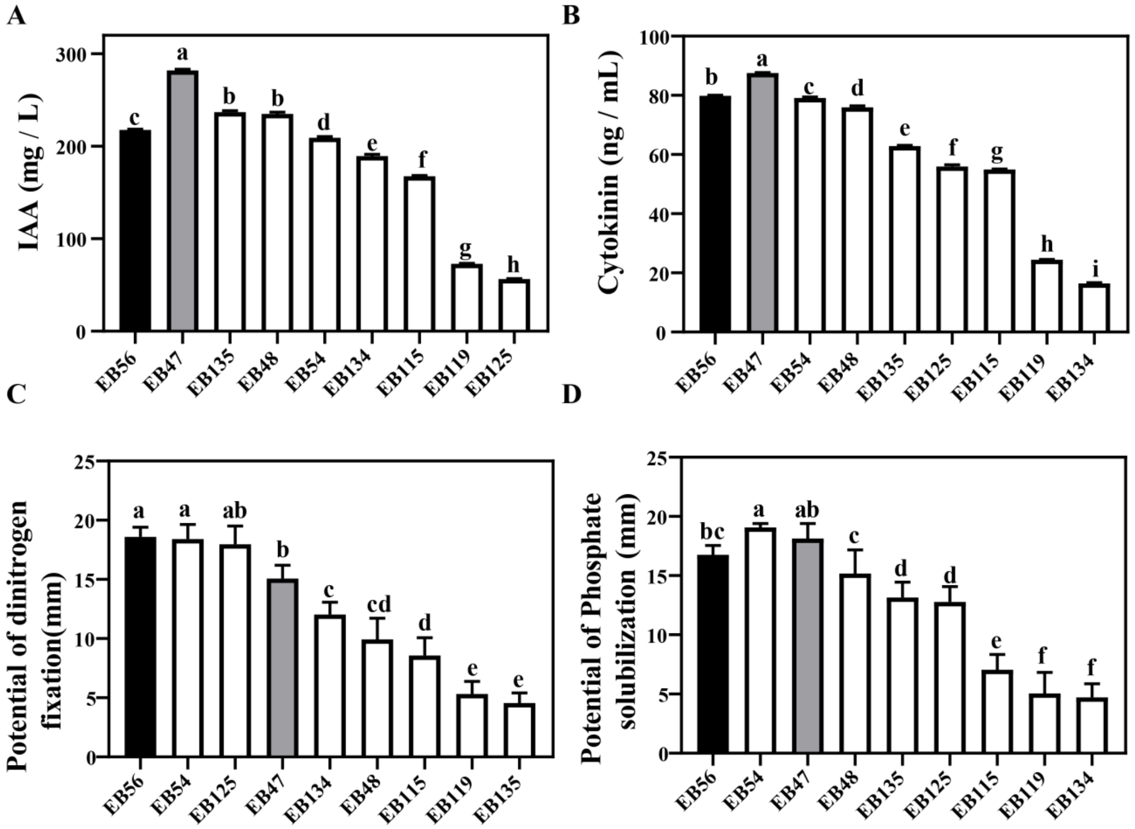

Supplementary Figure 5. Functional detection of selected isolates from genera consistent with keystones and biomarkers. Eight isolates (EB47, 48, 54, 115, 119, 125, 134, and 135) belonging to *Streptomyces* and one isolate (EB56) belonging to *Bradyrhizobium* were selected. Data of different functional indices: (A) IAA production; (B) Cytokinin production; (C) dinitrogen fixation; (D) Phosphorus solubilization. Error bars in columns are mean values ± SD (n=6). Different letters indicate significant differences according to one-way analysis of variance (ANOVA) with Tukey's HSD test ($P<0.05$).
