# Supplementary material for: Interspecific Neighbor Stimulates Peanut Growth Through Modulating Root Endophytic Microbial Community Construction
Source: Front Plant Sci. 2022 Mar 3;13:830666. doi: 10.3389/fpls.2022.830666 (PMC8928431; doi:10.3389/fpls.2022.830666)
Supplement: Supplementary file 6 [file Image_6.PDF]

Supplementary Information

Supplementary Figures

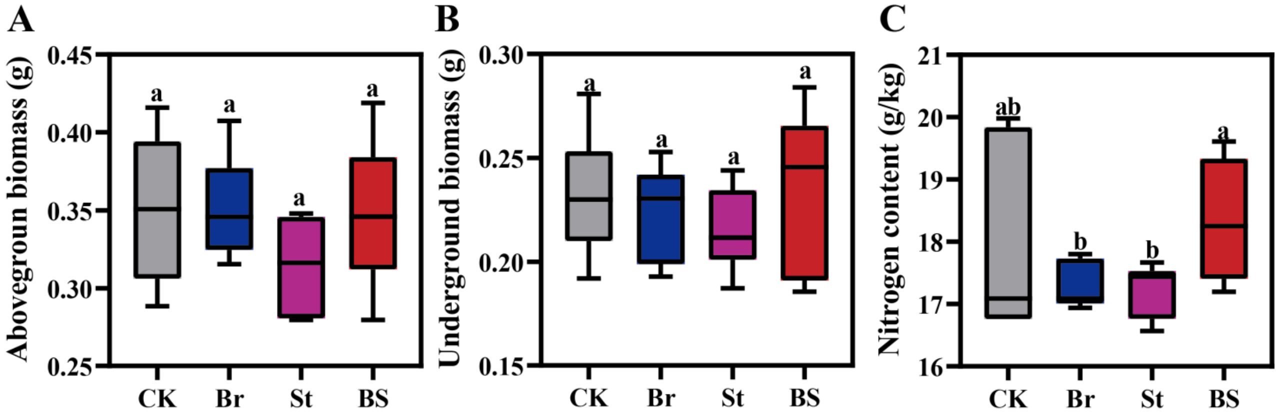

Supplementary Figure 6. Peanut physiological characteristics under exogenous inoculation of bacterial isolates. Data of different physiological indices: (A) Peanut aboveground biomass; (B) Peanut underground biomass; (C) Nitrogen content. CK, Control; Br, peanut seedlings inoculated with *Bradyrhizobium* EB56; St, peanut seedlings inoculated with *Streptomyces* EB47; BS, peanut seedlings inoculated with EB56 and EB47. Error bars in box are mean values ± SD (n=6). Different letters indicate significant differences according to one-way analysis of variance (ANOVA) with Tukey's HSD test (P<0.05).
