# Supplementary material for: Interspecific Neighbor Stimulates Peanut Growth Through Modulating Root Endophytic Microbial Community Construction
Source: Front Plant Sci. 2022 Mar 3;13:830666. doi: 10.3389/fpls.2022.830666 (PMC8928431; doi:10.3389/fpls.2022.830666)
Supplement: Supplementary file 7 [file Table_1.DOCX]

***Supplementary Information***

**Supplementary Tables**

**Supplementary Table 1.** We used the Permutational multivariate analysis of variance (PERMANOVA/ANOSIM) based on Bray-Curtis distance to test the effect of cropping systems on the community structure of plant root^*^.

| **Group** | **PERMANOVA** | | | **ANOSIM** | |
| --- | --- | --- | --- | --- | --- |
|  | **F** | **R^2^** | ***P*** | **R** | ***P*** |
| MMmr vs MPmr | 8.22 | 0.51 | **0.005** | 0.89 | **0.003** |
| MMmr vs MPpr | 15.28 | 0.66 | **0.006** | 0.99 | **0.005** |
| MMmr vs PPpr | 9.45 | 0.54 | **0.008** | 0.96 | **0.003** |
| MPmr vs MPpr | 10.11 | 0.56 | **0.012** | 0.97 | **0.002** |
| MPmr vs PPpr | 6.37 | 0.44 | **0.006** | 0.86 | **0.003** |
| MPpr vs PPpr | 9.39 | 0.54 | **0.004** | 0.87 | **0.003** |

**P* values with bolding indicated the significant differences between treatments.
