# Supplementary material for: Interspecific Neighbor Stimulates Peanut Growth Through Modulating Root Endophytic Microbial Community Construction
Source: Front Plant Sci. 2022 Mar 3;13:830666. doi: 10.3389/fpls.2022.830666 (PMC8928431; doi:10.3389/fpls.2022.830666)
Supplement: Supplementary file 8 [file Table_2.DOCX]

***Supplementary Information***

**Supplementary Tables**

**Supplementary Table 2.** The relative abundances (%) of differential bacterial phyla in peanut and maize root *.

| **Phyla** | **The relative abundance in 16S rRNA (%)** | | | |
| --- | --- | --- | --- | --- |
|  | **MMmr** | **MPmr** | **PPpr** | **MPpr** |
| *Actinobacteria* | 34.29±0.13b | 25.97±0.07bc | 21.01±0.11c | 52.22±0.06a |
| *Gammaproteobacteria* | 29.27±0.11a | 33.60±0.04a | 36.33±0.13a | 14.40±0.02b |
| *Bacteroidetes* | 16.70±0.02ab | 16.73±0.05ab | 20.74±0.05a | 13.44±0.04b |
| *Alphaproteobacteria* | 14.67±0.01a | 14.03±0.01a | 16.73±0.02a | 13.89±0.04a |
| *Patescibacteria* | 1.35±0.01a | 2.03±0.01a | 1.81±0.01a | 1.99±0.01a |
| *Deltaproteobacteria* | 0.84±0.01b | 2.57±0.01a | 0.43±0.01b | 0.59±0.01b |
| *Acidobacteria* | 0.22±0.01bc | 0.55±0.01a | 0.11±0.01c | 0.38±0.01ab |
| *Chloroflexi* | 0.14±0.01b | 0.66±0.01a | 0.08±0.01b | 0.16±0.01b |
| *Firmicutes* | 0.12±0.01b | 0.14±0.01b | 0.32±0.01a | 0.13±0.01b |
| *Gemmatimonadetes* | 0.07±0.01b | 0.24±0.01a | 0.08±0.01ab | 0.21±0.01ab |

* Different lowercase letters indicate significant differences (*P* < 0.05) across cropping systems, as revealed by ordinary one-way analysis of variance (one-way ANOVA) with Tukey’s honest significant difference (HSD) test. PPpr, peanut root microbiota in monocropping treatment; MPpr, peanut root microbiota in intercropping treatment; MMmr, maize root microbiota in monocropping treatment; MPmr, maize root microbiota in intercropping treatment.
