# Supplementary material for: Interspecific Neighbor Stimulates Peanut Growth Through Modulating Root Endophytic Microbial Community Construction
Source: Front Plant Sci. 2022 Mar 3;13:830666. doi: 10.3389/fpls.2022.830666 (PMC8928431; doi:10.3389/fpls.2022.830666)
Supplement: Supplementary file 9 [file Table_3.DOCX]

***Supplementary Information***

**Supplementary Tables**

| **Networks** | **ID** | **OTU** | **Phylum** | **Genus** | **Topological**  **role** | ***Zi***  **value** | ***Pi***  **value** |
| --- | --- | --- | --- | --- | --- | --- | --- |
| PPpr | g_Novosphingobium | OTU114 | *Gammaproteobacteria* | *Novosphingobium* | Module hub | 2.71 | 0 |
| MPpr | g_ Streptomyces | OTU454 | *Actinobacteria* | *Streptomyces* | connector | 0 | 0.65 |
|  | g_ Actinospica | OTU1222 | *Actinobacteria* | *Actinospica* | connector | -0.59 | 0.66 |
|  | g_ Bradyrhizobium | OTU3045 | *Alphaprotebacteria* | *Bradyrhizobium* | connector | 0 | 0.72 |
|  | g_ Amycolatopsis | OTU223 | *Actinobacteria* | *Amycolatopsis* | connector | -1.32 | 0.63 |

**Supplementary Table 3.** Nodes identified as module hubs and connectors in the network of monocroped and intercropped peanut roots.

* PPpr, peanut root microbiota in monocropping treatment; MPpr, peanut root microbiota in intercropping treatment.
