# Supplementary material for: Interspecific Neighbor Stimulates Peanut Growth Through Modulating Root Endophytic Microbial Community Construction
Source: Front Plant Sci. 2022 Mar 3;13:830666. doi: 10.3389/fpls.2022.830666 (PMC8928431; doi:10.3389/fpls.2022.830666)
Supplement: Supplementary file 10 [file Table_4.DOCX]

***Supplementary Information***

**Supplementary Tables**

**Supplementary Table 4.** Information of selected bacterial isolates from genera (*Bradyrhizobium* and *Streptomyces*) that was consistent with keystone organisms in the intercropped peanut root microbial network.

| **ID** | **Length (bp)** | **Genus** | **Phylum** | **Species match in NCBI** | **Matching rate (identity %)**  **in NCBI** |
| --- | --- | --- | --- | --- | --- |
| **Important genera in MPpr network** | | | | | |
| OTU3045  OTU454 | 378  379 | *Bradyrhizobium*  *Streptomyces* | *Alphaprotebacteria*  *Actinobacteria* | *Bradyrhizobiaceae bacterium*  *Streptomyces parvulus* | 97  97 |
| **Isolates that are affiliated with *Bradyrhizobium* and *Streptomyces*** | | | | | |
| EB56  EB47  EB135  EB48  EB54  EB134  EB115  EB119  EB125 | 1056 | *Bradyrhizobium* | *Alphaprotebacteria* | *Bradyrhizobium japonicum* | 99 |
|  | 906 | *Streptomyces* | *Actinobacteria* | *Streptomyces sp.* | 100 |
|  | 845  1107  887  846  857  801  846 | *Streptomyces*  *Streptomyces*  *Streptomyces*  *Streptomyces*  *Streptomyces*  *Streptomyces*  *Streptomyces* | *Actinobacteria*  *Actinobacteria*  *Actinobacteria*  *Actinobacteria*  *Actinobacteria*  *Actinobacteria*  *Actinobacteria* | *Streptomyces caviscabies*  *Streptomyces fabae*  *Streptomyces ambofaciens*  *Streptomyces olivaceus*  *Streptomyces nodosus*  *Streptomyces ferralitis*  *Streptomyces avellaneus* | 99  98  97  100  99  99  99 |
