# Supplementary material for: Interspecific Neighbor Stimulates Peanut Growth Through Modulating Root Endophytic Microbial Community Construction
Source: Front Plant Sci. 2022 Mar 3;13:830666. doi: 10.3389/fpls.2022.830666 (PMC8928431; doi:10.3389/fpls.2022.830666)
Supplement: Supplementary file 11 [file Table_5.DOCX]

***Supplementary Information***

**Supplementary Tables**

| **Group** | **PERMANOVA** | | | **ANOSIM** | |
| --- | --- | --- | --- | --- | --- |
|  | **F** | **R^2^** | ***P*** | **R** | ***P*** |
| CK vs Br | 0.86 | 0.10 | 0.49 | 0.01 | 0.43 |
| CK vs St | 1.57 | 0.16 | 0.23 | 0.09 | 0.17 |
| CK vs BS | 0.80 | 0.09 | 0.58 | 0.07 | 0.23 |
| Br vs St | 1.10 | 0.12 | 0.36 | 0.02 | 0.45 |
| Br vs BS | 0.63 | 0.07 | 0.73 | 0.06 | 0.60 |
| St vs BS | 1.52 | 0.16 | 0.20 | 0.04 | 0.27 |

**Supplementary Table 5.** Permutational multivariate analysis of variance (PERMANOVA /ANOSIM) based on Bray-Curtis distance to test the effect of exogenous bacterial inoculation effect on the composition of peanut root endophytic microbiota.
